# Charing Cross Hospital

**Published:** 1916-04-01

**Authors:** 


### Charing Cross Hospital.

### ANNUAL GENERAL COURT OF GOVERNORS.

The ninety-fifth annual Court of the hospital was held in the Board-room on Thursday, March 23, the chair, in the unavoidable absence of the Treasurer, the Earl of Lonsdale, being occupied by Mr. George Verity, J.P., the chairman of the hospital.

The Chairman, in moving the adoption of the report and accounts, said that the first time he presided at a Court the hospital had 140 beds; now there were 300, and the building was at length being used to its utmost capacity. With the increase in ward capacity, improvement in equipment and general efficiency could also be claimed, and the work altogether must be of great value to the community. The work foreshadowed in the previous report had been almost completed; the engine-room and electric-lighting installation had been remodelled, and the equipment of the *x*-ray department brought quite up to date. It was hoped in the near future to increase the accommodation and comfort of the nurses' quarters. The hospital mortgage had been reduced in fifteen years from £85,000 to £59,000, £9,000 of which was in the Sinking Fund, and any legacies not specially earmarked were applied to reduction of the capital debt. As the hospital supplied 747,000 meals per annum, the size of the housekeeping problem would be appreciated. The King's Hospital Fund Statistics showed that the cost per bed was the lowest but one of any hospital in the Metropolitan area. The Chairman made reference to the death of Lord Kilmorey, whose influence greatly helped the hospital when it was at a low ebb; also to the decease of Colonel Boyd, the senior surgeon, and other prominent workers. Owing to the necessarily darkened streets, the casualty list had been exceptionally heavy, and nothing could exceed the promptness and willingness of honorary and ordinary staffs in meeting the sudden calls upon the resources of the casualty department. He expressed the thanks of the Board for the seventeen months' unpaid service of Dr. Dickinson as resident medical officer. In connection with the tuberculosis dispensary, the attendances had been over 2,500 per annum, and this work would probably grow until it became an important integral part of the hospital's activities. They had been asked to co-operate with the Westminster Town Council in this matter, but the agreement had not been ratified, as the proposed remuneration was much too meagre. There was nothing to prevent the City Fathers establishing a centre of their own, with high salaries, though there did not seem any need for such expensive overlapping. He quite expected to be able to report next year that the hospitals in the neighbourhood were doing the actual work, and that that work had greatly increased.

Business of a formal character was followed by a resolution of gratitude to H.R.H. Princess Louise for the interest she had at all times displayed in the work of the institution. Reference was made by Dr. James Galloway, senior physician, to the timely and keen interest manifested by H.R.H. in 1911, a critical time for the hospital, and still more so for the medical school. Owing to the efforts at that date, the amalgamation foreshadowed eighty years ago was accomplished, the medical schools of Charing Cross Hospital and King's College now marched hand-in-hand, and the greatest benefit had ensued to both.

